# Contemporary approaches to treat people with hemophilia: what’s new and what’s not?

**DOI:** 10.1016/j.rpth.2025.102696

**Published:** 2025-01-31

**Authors:** Leonard A. Valentino, Maria E. Santaella, Samantha A. Carlson, Michael Recht

**Affiliations:** 1 Hemophilia and Thrombophilia Center, Rush University Medical Center, Chicago, Illinois, USA; 2 Research Department, National Bleeding Disorders Foundation, New York, New York, USA; 3 Department of Pediatrics, Hematology/Oncology, Yale School of Medicine, New Haven, Connecticut, USA

**Keywords:** health equity, hemophilia, novel therapies, prophylaxis, psychosocial, quality of life, shared decision-making

## Abstract

The care of people with hemophilia with access to treatment has evolved over the past 70 years, with an average life expectancy like unaffected peers. For people with hemophilia living in low- and middle-income countries, the same is not true because of the lack of access to diagnosis and treatment. It is imperative to close gaps in care that exist throughout the world.

Here, we provide a narrative review of hemophilia and the treatments available to people with hemophilia A and B with the goal of achieving a hemophilia-free state. We aim to provide information on what is new and what gaps remain that preclude equitable outcomes for everyone with hemophilia.

Information on the current state of hemophilia care and outcomes, the products available for the treatment of people with hemophilia, comprehensive interdisciplinary care of people with hemophilia, and the remaining gaps in care for people with hemophilia were assembled by the authors using relevant literature.

Research must focus on preventing all bleeding, and new approaches to detect joint bleeding are needed. Training on and implementation of comprehensive interdisciplinary care is needed to elevate the standards of care in low- and middle-income countries. The development and introduction of improved factor replacement and nonfactor products, such as second-generation bispecific monoclonal antibodies and targeted inhibitors of the anticoagulant mechanisms along with genetic therapies, have the possibility of normalizing hemostasis and achieving health equity for people with hemophilia.

Improved outcomes and, ultimately, health equity, can only be realized if diagnosis, education, and care are accessible to everyone living with hemophilia worldwide.

## Essentials

• Advances in care improve outcomes, but many with hemophilia still face morbidity and mortality.
• Equitable access to care and treatment is essential for a hemophilia-free mindset globally.
• Research must prioritize preventing all bleeding in new hemophilia therapies.
• Health equity for hemophilia requires accessible diagnosis, education, and care worldwide.

## 1 Introduction

The approach to treating people with hemophilia living in countries without constrained resources has evolved considerably over the past 7 decades with the advent of advanced therapeutics [1] and the implementation of comprehensive, integrated care [2]. Unfortunately, for people with hemophilia living in low- and middle-income countries (L/MIC) where resources are constrained, the same therapeutic options are not readily accessible, resulting in inferior outcomes. Despite resource availability, people-centered medicine has emerged as a concept advanced by the World Health Organization and numerous professional societies to address concerns of compartmentalization of knowledge, fragmentation of services, and neglect of patients’ concerns, needs, and values [3,4]. This review will discuss the technological changes moving from standard half-life (SHL) replacement products to extended half-life (EHL) factor replacement products and the introduction of the first nonfactor therapy, a bispecific monoclonal antibody and molecules that increase thrombin generation by targeting specific inhibitors of the coagulation system as well as the introduction of genetic medicines to treat people with hemophilia A and B and how the delivery of integrated comprehensive care has facilitated a people-centric care approach [5] to enhance the health and well-being of people with hemophilia. Finally, we will provide insights into existing gaps in care and areas for further research and opportunities for innovation to reach the goal of health equity for all people with hemophilia, irrespective of where they live.

## 2 Overview of Hemophilia

Hemophilia is due to a defect in the body’s ability to generate thrombin and convert fibrinogen to fibrin, the basis of a stable clot [6]. Typically, after a breach in a blood vessel wall, small amounts of thrombin are generated in the initiation phase of coagulation either on the surface of subendothelial smooth muscle cells or fibroblasts. An amplification phase then occurs on the surface of activated platelets through the effects of activated factor (F)IX and FVIII, resulting in FXa generation and a subsequent burst of thrombin [7]. When the activity of FVIII (hemophilia A) or FIX (hemophilia B) is missing or defective, thrombin generation and clot formation are ineffective, resulting in a bleeding phenotype [8]. Thus, thrombin generation assays, if standardized and performed consistently, can potentially be used to predict bleeding and personalize treatment for people with hemophilia [9].

The severity of hemophilia has traditionally been defined by the residual amounts of FVIII or FIX activity in plasma (severe disease [<0.01 IU/mL], moderate [0.01-0.05 IU/mL], and mild [0.06-0.40 IU/mL]) [10]. Recently, more precise definitions of severity by bleeding tendency or phenotype (as the 2 do not always coincide) have been proposed [11].

The bleeding phenotype of people with hemophilia, as noted above, is primarily determined by the baseline factor activity measurable in plasma; however, variability in laboratory assay performance [12,13], along with other possible disease-modifying factors [14], may impact the phenotype of people with hemophilia, including the mutation in the coagulation factor gene, other genetic alterations, and polymorphisms in other genes of the hemostatic system (eg, FV Leiden or antithrombin) as well as genetic variability of inflammatory and immune response genes [15]. Environmental factors and other as yet not fully explored issues may include birth sex (most if not all of the studies have been performed only in males), levels of physical activity, and the use of other medications that may play a role in determining an individual’s bleeding tendency [14]. Finally, the tools and measures used to assess outcomes also play a role in defining the severity of the disease [16].

Bleeding in people with hemophilia may be defined clinically as having a severe phenotype when the first spontaneous bleeding event occurs before 6 months of age, spontaneous joint bleeding occurs before 2 years of age, unprovoked intracranial hemorrhage occurs at any age, or frequent or large spontaneous subcutaneous hematomas or at least 10 bleeding events per year are observed [17]. Bleeding into the joints is the most frequent severe manifestation of hemophilia [[18], [19], [20]] and leads to inflammatory and proliferative synovitis, which can be debilitating and lead to a decreased health-related quality of life (HRQoL) [21].

According to the Annual Global Survey produced by the World Federation of Hemophilia (WFH) for 2022 [22], 257,146 people with hemophilia (208,957 with hemophilia A) were reported from 125 countries, including 11,700 females. Based on the global population and the estimated prevalence of hemophilia at birth [23], this number of reported people with hemophilia (257,146) represents approximately 22% of the expected number of people with hemophilia (1,184,000) worldwide (see formulas 1 and 2), including about 282,266 with FVIII or FIX activity levels < 1% of normal. Even in a high-income country such as the United States of America (USA), the reported estimated number of people with hemophilia is only 56% (18,580 identified people with hemophilia based on the US Centers for Disease Control and Prevention Community Counts Registry [24] and 33,000 estimated people with hemophilia [23]), suggesting that access to diagnosis and care remains an issue even in high-income countries. This issue is further magnified when considering the number of women reported to have hemophilia, in which it is estimated that for one man with hemophilia, 2.7 to 5 potential carriers may be found in that family [25,26].

The WFH has published guidelines for the management of people with hemophilia [27] in which it is stated that individuals with a severe phenotype (including those traditionally defined as having moderate hemophilia but with a severe bleeding phenotype) should receive prophylaxis sufficient to prevent bleeding at all times. Over the past 50 years, the goals of hemophilia treatment have evolved from lifesaving (eg, preventing intracranial hemorrhage) to preventing chronic disabling joint disease, normalizing life, and health equity with unaffected peers [28]. This is in part due to the widespread implementation of integrated, comprehensive care [2] based on best practices in hemophilia care delivery [29] and marked progress in therapies available to prevent bleeding.

Formula1:4,000,000,000÷100,000=40,000×29.6=1,184,000

Formula2:257,146÷1,184,000×100=21.7%

Global population estimated to be 8 billion in 2022, of which 4 billion were males according to the International Database (https://www.census.gov/data-tools/demo/idb/#/dashboard)

Estimated prevalence of hemophilia at birth (per 100 000 males) is 24.6 cases for all severities of hemophilia A and 5.0 cases for all severities of hemophilia B, totaling 29.6 per 100,000 males [1].

257,146 PwH according to the WFH AGS 2022

## 3 Products Available for People with Hemophilia

There are 5 categories of products currently available or in clinical development for people with hemophilia to treat bleeding and, more importantly, to prevent bleeding if used as prophylaxis. These categories include SHL clotting factor concentrates; EHL clotting factor concentrates; 1 bispecific monoclonal antibody with regulatory authorization for the prevention of bleeding in people with hemophilia A and at least 2 others in clinical development; 1 rebalancing agent targeting tissue factor (TF) pathway inhibitor (TFPI) with regulatory authorization and a number of rebalancing agents in clinical development; and 3 gene therapy products, 1 for hemophilia A and 2 for hemophilia B with regulatory authorization and many more in different stages of development (Tables 1 and 2).

**Table 1:** Products available to treat people with hemophilia.

Product	Proper name	Manufacturer	Indication	Route of administration	Strengths (IU)	Storage
**Human plasma-derived concentrates that contain FVIII and von Willebrand factor to treat hemophilia A**						
Alphanate	Antihemophilic factor/von Willebrand factor complex (human)	Grifols Biologicals Inc	Control and prevention of bleeding in patients with hemophilia A or acquired FVIII deficiency. Surgical and/or invasive procedures in adult and pediatric patients with VWD in whom DDAVP is either ineffective or contraindicated. It is not indicated for patients with severe VWD (type 3) undergoing major surgery.	Intravenous, 5 or 10 mL of sterile water for injection	250, 500, 1000, and 1500 IU FVIII in a single-use vial	2 and 8 °C or may be stored at room temperature not to exceed 30 °C for up to 2 mo.
Koate-DVI	Antihemophilic factor (human)	Kedrion Biopharma	Control and prevention of bleeding episodes or in order to perform emergency and elective surgery in patients with hemophilia A (hereditary FVIII deficiency).	Intravenous, 5 or 10 mL of sterile water for injection	250, 500, and 1000 IU FVIII in a single-use vial	2 and 8 °C or may be stored at room temperature not to exceed 30 °C for up to 6 mo.
Humate P	Antihemophilic factor/von Willebrand factor complex (human)	CSL Behring GmbH	Hemophilia A–treatment and prevention of bleeding in adults. VWD–in adults and pediatric patients in the (1) treatment of spontaneous and trauma-induced bleeding episodes and (2) prevention of excessive bleeding during and after surgery.	Intravenous, 5, 10, or 15 mL of sterile water for injection	600 IU VWF:RCo and 250 IU FVIII; 1200 IU VWF:RCo and 500 IU FVIII; and 2400 IU VWF:RCo and 1000 IU FVIII per vial	Stored at temperatures up to 25 °C (77 °F) for 36 mo up to the expiry date.
**Human plasma-derived immunoaffinity-purified FVIII concentrates to treat hemophilia A**						
Hemofil M	Antihemophilic factor human, method M, monoclonal purified	Takeda Pharmaceuticals U.S.A., Inc	For the prevention and control of hemorrhagic episodes in hemophilia A.	Intravenous, 10 mL of sterile water for injection	250, 500, 1000, and 1700 IU vials	2 to 8 °C (36-46 °F) or at room temperature, not to exceed 30 °C (86 °F) until the expiry date.
**SHL recombinant products to treat hemophilia A**						
Advate	Antihemophilic factor (recombinant), plasma/albumin-free method	Takeda Pharmaceuticals U.S.A., Inc	Control and prevention of bleeding episodes in adults and children (0-16 y), perioperative management in adults and children (0-16 y), and routine prophylaxis to prevent or reduce the frequency of bleeding episodes in adults and children (0-16 y)	Intravenous, 2 or 5 mL of sterile water for injection	250, 500, 1000, 1500, 2000, and 3000 IU vials	2 to 8 °C and room temperature (up to 30 °C [86 °F]) for a period of up to 6 mo
NovoEight	Antihemophilic factor (recombinant)	Novo Nordisk	For adults, adolescents, and children with hemophilia A for the control and prevention of bleeding episodes, perioperative management, and routine prophylaxis to prevent or reduce the frequency of bleeding episodes.	Intravenous, 4 mL prefilled sodium chloride diluent in a syringe	250, 500, 1000, 1500, 2000, and 3000 IU	2 to 8 °C and room temperature (up to 30 °C [86 °F]) for a period of up to 12 mo
NUWIQ	Antihemophilic factor (recombinant)	Octapharma USA, Inc	Indicated in adults and children with hemophilia A for on-demand treatment and control of bleeding episodes, perioperative management of bleeding, and routine prophylaxis to reduce the frequency of bleeding episodes.	Intravenous, 2.5 mL prefilled syringe with water for injection	250, 500, 1000, and 2000 IU	2 to 8 °C and room temperature (up to 30 °C [86 °F]) for a period of up to 24 mo
Recombinate	Antihemophilic factor (recombinant)	Baxter Healthcare Corporation	Indicated in adults and children with hemophilia A for the prevention and control of hemorrhagic and perioperative management of patients with hemophilia A.	Intravenous, 5 mL of sterile water for injection	250, 500, 1000, 1500, and 2000 IU	2 to 8 °C and room temperature (up to 30 °C [86 °F])
Xyntha	Antihemophilic factor (recombinant), plasma/albumin free	Wyeth Pharmaceuticals, LLC	Indicated in adults and children with hemophilia A for on-demand treatment and control of bleeding episodes, perioperative management, and routine prophylaxis to reduce the frequency of bleeding episodes.	Intravenous, 4 mL prefilled syringe with 0.9% sodium chloride	250, 500, 1000, or 2000 IU.	2 to 8 °C and room temperature (up to 30 °C [86 °F]) for a period of up to 3 mo
**EHL recombinant products to treat hemophilia A**						
Adynovate	Antihemophilic factor, recombinant, PEGylated	Takeda Pharmaceuticals U.S.A., Inc	On-demand treatment and control of bleeding episodes, perioperative management, and routine prophylaxis to reduce the frequency of bleeding episodes.	Intravenous, 2 or 5 mL of sterile water for injection in a BAXJECT III system	250, 500, 750, 1000, 1500, 2000, or 3000 IU vials	2 to 8 °C and room temperature (up to 30 °C [86 °F]) for a period of up to 3 mo
Afstyla	Antihemophilic factor (recombinant), single chain	CSL Behring GmbH	On-demand treatment and control of bleeding episodes, routine prophylaxis to reduce the frequency of bleeding episodes, and perioperative management of bleeding.	Intravenous, 2.5 or 5 mL of sterile water for injection	250, 500, 1000, 1500, 2000, 2500, or 3000 IU	2 to 8 °C and room temperature (up to 25 °C [77 °F]) for a period of up to 3 mo
Altuviiio	Antihemophilic factor (recombinant), Fc-VWF-XTEN fusion protein-ehtl	Bioverativ Therapeutics Inc	Routine prophylaxis to reduce the frequency of bleeding episodes, on-demand treatment and control of bleeding episodes, and perioperative management of bleeding.	Intravenous, prefilled syringe with 3 mL of sterile water for injection	250, 500, 750, 1000, 2000, 3000, or 4000 IU	2 to 8 °C and room temperature (up to 30 °C [86 °F]) for a period of up to 6 mo
Eloctate	Antihemophilic factor (recombinant), Fc fusion protein	Biogen Idec, Inc	For adults and children with hemophilia A for (1) on-demand treatment and control of bleeding episodes, perioperative management, and routine prophylaxis to prevent or reduce the frequency of bleeding episodes.	Intravenous, prefilled syringe with 3 mL of sterile water for injection	250, 500, 750, 1000, 1500, 2000, or 3000 IU	2 to 8 °C and room temperature (up to 30 °C [86 °F]) for a period of up to 12 mo
Esperoct	Antihemophilic factor (recombinant), GlycoPEGylated-exei	Novo Nordisk, Inc	Adults and children with hemophilia A for control and prevention of bleeding, perioperative management, and routine prophylaxis to reduce the frequency of bleeding episodes.	Intravenous, MixPro (NovoNordisk) prefilled diluent syringe with 4 mL of 0.9% saline solution	500, 1000, 1500, 2000, and 3000 IU	2 to 8 °C and room temperature (up to 30 °C [86 °F]) for a period of up to 6 mo
Jivi	Antihemophilic factor (recombinant), PEGylated-aucl	Bayer Healthcare, Inc	Indicated for use in previously treated adults and adolescents (12 y of age and older) with hemophilia A (congenital FVIII deficiency) for on-demand treatment and control of bleeding episodes, perioperative management of bleeding, and routine prophylaxis to reduce the frequency of bleeding episodes.	Intravenous, prefilled diluent syringe with 2.5 mL of sterile water for injection	500, 1000, 2000, or 3000 IU	2 to 8 °C and room temperature (up to 30 °C [86 °F]) for a period of up to 6 mo
Kovaltry	Antihemophilic factor (recombinant), full length	Bayer HealthCare, LLC	For use in adults and children with hemophilia A for on-demand treatment and control of bleeding episodes, perioperative management of bleeding, and routine prophylaxis to reduce the frequency of bleeding episodes.	Intravenous, prefilled syringe with 2.5 or 5 mL of sterile water for injection	250, 500, 1000, 2000, or 3000 IU	2 to 8 °C and room temperature (up to 30 °C [86 °F]) for a period of up to 12 mo
**Recombinant humanized bispecific FIXa and FX-directed monoclonal antibody to treat hemophilia A**						
Hemlibra	emicizumab-kxwh	Genentech, Inc	Routine prophylaxis to prevent or reduce the frequency of bleeding episodes in adult and pediatric patients ages newborn and older with hemophilia A with or without FVIII inhibitors.	Subcutaneous injection	12 (0.4), 30 (1.0), 60 (0.4), 105 (0.7), 150 (1.0), and 300 (2.0 mL) mg per vial	36 to 46 °F (2-8 °C)
**Gene therapy products to treat hemophilia A**						
Roctavian	valoctocogene roxaparvovec-rvox	BioMarin Pharmaceutical Inc	Treatment of adults with severe hemophilia A (congenital FVIII deficiency with FVIII activity < 1 IU/dL) without preexisting antibodies to adeno-associated virus serotype 5 detected by an FDA-approved test.	Intravenous injection of 6 × 10e13 vg per kg of body weight	2 × 10e13 vg per mL, each vial contains an extractable volume of not less than 8 mL (16 × 10e13 vg).	Frozen at ≤−60 °C (−76 °F)
**Human plasma-derived coagulation FIX concentrates to treat hemophilia B**						
AlphaNine SD	Coagulation FIX (human)	Grifols Biologicals LLC	Prevention and control of bleeding in patients with FIX deficiency due to hemophilia B.	Intravenous, 10 mL of sterile water for injection	500, 1000, or 1500 IU	2 and 8 °C (36 and 46 °F) for 3 y up to the expiry date
Mononine	Coagulation FIX (human) monoclonal antibody purified	CSL Behring, LLC	Prevention and control of bleeding in FIX deficiency, also known as hemophilia B or Christmas disease.	Intravenous, 5 or 10 mL of sterile water for injection	500 or 1000 IU	2 to 8 °C (36-46 °F)
**SHL products to treat hemophilia B**						
Benefix	Coagulation FIX (recombinant)	Pfizer	Treatment of adults and children with hemophilia B for on-demand treatment and control of bleeding episodes or perioperative management of bleeding. Patients 16 y of age and older with hemophilia B for routine prophylaxis to reduce the frequency of bleeding episodes.	Intravenous	250, 500, 1000, 2000, or 3000 IU per vial	Room temperature or under refrigeration at a temperature of 2 to 30 °C (36-86 °F)
Ixinity	Coagulation FIX (recombinant)	Medexus Pharma, Inc	On-demand treatment and control of bleeding episodes, perioperative management, and routine prophylaxis to reduce the frequency of bleeding episodes.	Intravenous, 5 or 2 mL of sterile water for injection	250, 500, 1000, 1500, 2000, or 3000	250 IU strength only; store at 2 to 8 °C, other strengths store at 2 to 25 °C
Rixubis	Coagulation FIX (recombinant), nonacog gamma	Takeda Pharmaceuticals U.S.A., Inc	For adults and children with hemophilia B, for (1) control and prevention of bleeding episodes, (2) perioperative management, and (3) routine prophylaxis.	Intravenous, 5 mL of sterile water for injection	250, 500, 1000, 2000, or 3000 IU per vial	2 to 8 °C (36-46 °F) for up to 24 mo and room temperature not to exceed 30 °C (86 °F) for up to 12 mo within the 24-mo time period.
**EHL recombinant products to treat hemophilia B**						
Alprolix	Coagulation FIX (recombinant), Fc fusion protein	Sanofi	Adults and children with hemophilia B for on-demand treatment and control of bleeding episodes, perioperative management of bleeding, and routine prophylaxis to reduce the frequency of bleeding episodes.	Intravenous, 5 mL of sterile water for injection	250, 500, 1000, 2000, 3000, or 4000 IU per vial	2 to 8 °C (36-46 °F) or at room temperature, not to exceed 30 °C (86 °F) for a single period of up to 6 mo within the expiration date
IDelvion	Coagulation FIX (recombinant), albumin fusion protein	CSL Behring, LLC	Children and adults with hemophilia B for on-demand treatment and control of bleeding episodes, perioperative management of bleeding, and routine prophylaxis to reduce the frequency of bleeding episodes.	Intravenous, 2.5 or 5 mL of sterile water for injection	250, 500, 1000, 2000, or 3500 IU per vial	In refrigerator or at room temperature 2 to 25 °C (36-77 °F)
Rebinyn	Coagulation FacIX (recombinant), GlycoPEGylated	NovoNordisk, Inc	Adults and children with hemophilia B for on-demand treatment and control of bleeding episodes and perioperative management of bleeding.	Intravenous, 4 mL of histidine diluent for injection	500, 1000, or 2000, IU per vial	36 to 46 °F (2-8 °C) for up to 24 mo from the date of manufacture until the expiration date stated on the label and may be stored at room temperature not to exceed 86 °F (30 °C) for up to 6 mo within the 24-mo time period.
**Gene therapy products to treat hemophilia B**						
Hemgenix	etranacogene (dezaparvovec-drlb) suspension	CSL Behring, LLC	Adults with hemophilia B who currently use FIX prophylaxis therapy, have a current or historical life-threatening hemorrhage, or have repeated serious spontaneous bleeding episodes.	Intravenous, 1 × 10e13 gc/mL	2 × 10e13 gc/kg of body weight	2 to 8 °C (36-46 °F).
BEQVEZ	fidanacogene (elaparvovec-dzkt) injection	Pfizer, Inc	Adults with moderate to severe hemophilia B (congenital FIX deficiency) who currently use FIX prophylaxis therapy, have a current or historical life-threatening hemorrhage, or have repeated serious spontaneous bleeding episodes and do not have neutralizing antibodies to adeno-associated virus serotype Rh74var (AAVRh74var) capsid as detected by an FDA-approved test.	Intravenous, 1 × 10e13 vg/mL, and each vial contains an extractable volume of 1 mL	5 × 10e11 vg/kg of body weight	Frozen (−100 °C to −60 °C [−148 °F to −76 °F])

DDAVP, desmopressin; EHL, extended half-life; FDA, US Food and Drug Administration; FIX/FVIII/FX, factor IX/VIII/X; FIXa, activated factor IX; gc, genome copies; IU, International Units; SHL, standard half-life; vg, vector genomes; VWD, von Willebrand disease; VWF:RCo, von Willebrand Factor Ristocetin Cofactor.

**Table 2:** Potential research questions that might be addressed by the USA National Research Blueprint.

**Diagnosis and monitoring**
Are there new tools in development to lower the diagnostic burden of people with hemophilia?
Can the identification of people with hemophilia be improved to increase diagnosis and access to care?
How to define the severity of hemophilia according to the lived experience?
How can thrombin generation assays be standardized to facilitate clinical use?
How to optimally measure and utilize thrombin generation to personalize hemophilia treatment?
What tools are needed to monitor the novel therapies that result in very low rates of bleeding?
**Care and treatment**
How to implement an SDM approach to make optimal use of the choices people with hemophilia have available to them to prevent bleeding?
Is there a role for SHL products in the current treatment landscape, and if so, where?
How can the use of EHL products be further optimized to improve the outcomes of people with hemophilia?
How to optimize the outcomes, especially joint outcomes, for people with hemophilia A who choose to use a bispecific monoclonal antibody?
What is the role of gene therapy in the current treatment landscape for people with hemophilia A?
How will the TFPI molecules and fitusiran be incorporated into the treatment decision-making?
Is there a clinical advantage to eptacog beta?
How to determine the safety of gene therapy for people with hemophilia?
What additional services can support mental health and the psychosocial impact for people with hemophilia?
How do we evaluate the success of contemporary treatment of people with hemophilia?
How can telehealth services facilitate optimal outcomes for people with hemophilia?
**Education**
What are the educational needs of healthcare providers and people with hemophilia in terms of novel therapies?
What new educational techniques might be introduced to facilitate SDM?
Can simulation be used to enhance patient education about bleeding disorders?
Are there opportunities to increase the use of telehealth services to expand access to care?
**Biology**
Why is it that bleeding into a joint results in joint damage and arthropathy?
What role does extravascular FIX play in hemostasis?
How do bleeding disorders manifest in women, and what outcome measures should be used to assess women?

FIX, factor IX; SDM, shared decision-making; TFPI, tissue factor pathway inhibitor.

There are several SHL products authorized by the US Food and Drug Administration (FDA) for the treatment and prevention of bleeding events in people with hemophilia A or B (Table 1). A comprehensive listing of all products available in the USA can be found on the National Bleeding Disorders Foundation (NBDF) Medical and Scientific Advisory Council website [30] and globally may be found on the WFH Online Registry of Clotting Factor Concentrates [31]. Products are either derived from plasma, virally inactivated and concentrated, or manufactured by recombinant technology and undergo additional purification steps, including viral inactivation.

The SHLs are characterized by a circulating half-life in the plasma similar to naturally occurring FVIII or FIX, 8 to 14 hours for FVIII and 12 to 18 hours for FIX [32]. The pharmacokinetic profile (Figure) has a “saw-tooth” pattern with a rapid rise and fall, necessitating frequent dosing every 2 to 3 days for FVIII and 3 to 5 days for FIX concentrates. The EHL FVIII concentrates have only marginal prolongation of FVIII half-life in the plasma, while EHL FIX concentrates demonstrate more substantial prolongations, allowing for weekly and even less frequent dosing intervals for those with hemophilia B. In 2023, a truly EHL product (antihemophilic factor [recombinant], Fc-VWF-XTEN [Sanofi Pharmaceuticals] fusion protein-ehtl) for use by people with hemophilia A received regulatory authorization allowing for weekly or less frequent dosing [33]. The pharmacokinetic profile of this molecule more closely resembles the EHL FIX products. One nonfactor therapy is currently authorized in the USA for people with hemophilia A, a bispecific monoclonal antibody binding to activated FIX and FX to bypass the requirement for FVIIIa in the process of thrombin generation. This molecule has a half-life similar to that of immunoglobulin G and can be dosed subcutaneously at weekly or up to monthly intervals, maintaining hemostatic efficacy and decreasing the treatment burden for people with hemophilia A. Three gene therapies, 1 for hemophilia A (valoctocogene roxaparvovec/Roctavian) and 2 for hemophilia B (etranacogene dezaparvovec/Hemgenix and fidanacogene elaparvovec-dkzt /Beqvez) received FDA marketing authorization in 2022, 2023, and 2024, respectively. In the Figure, the durability of hemophilia A gene therapy is limited in some recipients, while in others, it persists, similar to the responses observed in hemophilia B.

**Figure:**
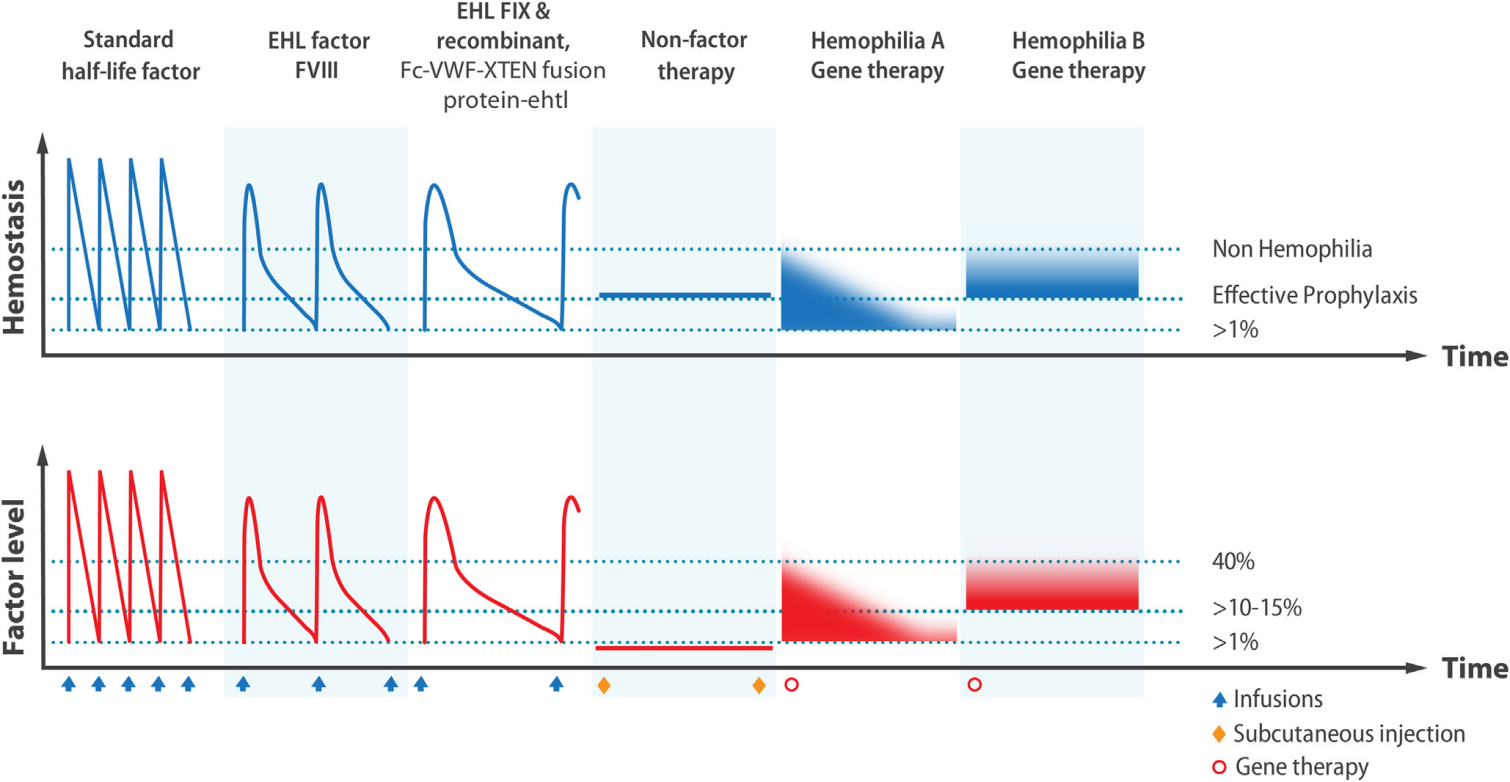
Five categories of therapeutics are available in the clinic or are in development to treat people with hemophilia, including standard half-life and extended half-life (EHL) factor (F)VIII and FIX replacement products, nonfactor therapies, and gene therapy for hemophilia A and B. The pharmacokinetic profiles of standard half-life and EHL products, along with their characteristic peaks and troughs of factor levels (*red*) and hemostatic effect (*blue*), are shown in the lower and upper profiles. Trough FVIII or FIX levels have historically been targeted to be above 1 IU/dL (1%), but effective prophylaxis and suppression of bleeding likely only is achieved when the trough level is maintained at substantially higher (>10%-15%) levels. The attainment of hemostasis in the nonhemophilic range is currently only achievable following successful gene therapy. The uncertain durability of hemophilia A gene therapy is shown by the parabolic curve, while that of hemophilia B gene therapy appears to be stable and durable. The frequency of intravenous infusions (blue) and subcutaneous injections (red) of the products is shown under the lower profile by the up arrows (), and the one-time infusion of a gene therapy product is shown by an open circle ().

The SHL FVIII products are considered in 3 generations based on their manufacturing processes and final formulations. The first-generation products contain albumin or other animal or human proteins throughout the manufacturing process and in the final formulation. Second-generation products have animal or human proteins purified from the final formulation but may have animal or human proteins in the manufacturing process. Third-generation products are devoid of animal and human proteins in both the manufacturing process and the final formulation. These molecules demonstrate similar efficacy in terms of the control of bleeding events and prophylaxis and have similar pharmacokinetic properties. There are differences in the production cell lines, the infusion volumes, and the rate of inhibitor development in previously untreated patients. Challenges with the SHL FVIII and FIX products include the frequency of infusions, their intravenous route of administration, inhibitor development, increased treatment burden, their high cost, and the inability to fully prevent bleeding.

The EHL products are produced by fusion with albumin or the Fc portion of immunoglobulin, by the addition of polyethylene glycol, or by synthesis as a single chain, in the case of FVIII. These molecules are similar in terms of their pharmacokinetics, efficacy, intravenous route of administration, and inhibitor rates. They differ in their production cell lines, infusion volumes, vial sizes, storage requirements, and, in the case of EHL FIX, the extravascular tissue distribution. Challenges with EHL products include the frequency of infusions (although improved from SHLs), their intravenous route of administration, inhibitor development, treatment burden, and cost.

Clinical outcomes with factor replacement therapy, whether with SHL or EHL products, include their safety, effectiveness, and burden of treatment. Currently available factor products are free from contamination with infectious agents but do carry a risk for inhibitor development. They reduce but do not eliminate all bleeding, including joint bleeding. An example of this comes from the Antihemophilic Factor Hemophilia A Outcome Database (AHEAD) study, which examined the effectiveness and safety of prophylaxis with the SHL FVIII, octocog alfa [34]. In almost 600 participants receiving prophylaxis, 44% to 66% failed to achieve a zero annualized bleeding rate (ABR) over the study follow-up period. Another example of real-world data demonstrating the reduction but not elimination of bleeding following a switch from recombinant (r)FVIII to recombinant FVIII Fc fusion protein (rFVIIIFc) comes from Taiwan, in which switching to rFVIIIFc prophylaxis led to uniform reductions in ABRs but not total suppression of bleeding [35]. The reduced frequency of intravenous injections was associated with less caregiver burden and emotional distress [36], but EHL products may increase the cost of prophylaxis and increase the financial burden on people with hemophilia, their families, and the health care system [37]. Since these products do not eliminate the possibility of joint bleeding, arthropathy and associated pain remain a problem with factor replacement therapy [38,39].

Bispecific monoclonal antibody therapy has demonstrated efficacy in children, adolescents, and adults with hemophilia A with and without inhibitors directed to FVIII [[40], [41], [42]]. In a review of the long-term outcomes of more than 400 participants in the Hemophilia A Clinical Trials Evaluating Emicizumab Prophylaxis (HAVEN) clinical development program, Callaghan et al. [42] demonstrated that 82% experienced zero treated bleeds and 98% had 3 or fewer treated bleeding events. However, the 95% CIs were broad, resulting in some participants having a calculated ABR as high as 5 to 6. The calculated average annualized joint bleeding rates from this analysis were approximately 0.5, but again, the 95% CI was as high as 4.8 after week 25 of the study period. Warren et al. [43] reported wide variability in response among people with hemophilia A treated with emicizumab in Colorado. Among 68 children and adults who switched to emicizumab, 37% (25/68) experienced zero bleeds, but 7% (5/68) experienced more than 8 bleeding episodes per year, reported subjectively by people with hemophilia or their caregivers. Data from a single center in Bangladesh demonstrated a remarkable reduction in bleeding among people with hemophilia with and without inhibitors to FVIII, resulting in improvements in joint health scores; however, 44% still experienced some bleeding (ABR > 0) [44]. Recent data reporting the long-term follow-up from the HAVEN-3 and -4 trials of emicizumab demonstrated a similar rate of bleeding: the ABRs (95% CI) for all participants were 1.4 (1.10-1.74) for treated bleeds and 0.9 (0.66-1.15) for treated joint bleeds [45]. This outcome is not dissimilar from the results of Oldenburg [38], who demonstrated an ABR of 0.5 with FVIII prophylaxis in a highly adherent cohort of people with hemophilia A, but these individuals still had evidence of long-term joint damage despite this low rate of joint bleeding. Real-world evidence demonstrates similar rates of bleeding, including joint bleeding, in people with hemophilia who received emicizumab prophylaxis [[46], [47], [48], [49], [50], [51], [52], [53], [54]] as those who received prophylaxis with factor concentrates. Highlighting this point, Escobar et al. [52] performed a comparison study in 131 people with hemophilia A without inhibitors before and after switching to emicizumab and demonstrated no significant reduction in ABRs, suggesting that the change in therapy did provide incremental benefit. Given this information, the long-term outcomes, especially in terms of joint outcomes, will need to be established for those with hemophilia A without inhibitors who receive emicizumab on a long-term basis. An interventional, prospective, randomized phase 4 controlled trial is underway to examine the joint health of children with hemophilia A after receiving 1 of 2 EHL FVIII products or emicizumab for 1 year (NCT04690322). Longer follow-up studies will likely be needed to determine the risks and benefits of this approach.

Novel products currently available or in clinical development include efanesoctocog alpha, additional high-potency bispecific monoclonal antibodies, more potent factor molecules, rebalancing agents, and gene therapies.

Efanesoctocog alpha is a rFVIII protein with 3 half-life extension technologies designed to overcome the half-life ceiling imposed by the naturally occurring von Willebrand factor. It combines the Fc fusion technology with the incorporation of the D’D3 region of von Willebrand factor and XTEN polypeptides to allow weekly and possibly less frequent dosing while maintaining FVIII trough levels above 3 to 5 IU/dL [55], as recommended by the WFH [27].

Two next-generation bispecific monoclonal antibodies are in clinical development. NXT007 demonstrates similar hemostatic activity compared with emicizumab but at a 30-fold lower concentration [56]. This molecule may be prone to the development of antidrug antibodies, which developed in 9 of 30 recipients, although none of these antidrug antibodies were inhibitory. The second next-generation bispecific monoclonal antibody is denecimig or Mim8. This molecule appears well tolerated, and there were no severe treatment-emergent adverse events in healthy adult recipients [57]. The pharmacokinetic and pharmacodynamic properties of Mim8 were consistent with dose proportionality, allowing for once-monthly dosing. Preliminary results from a phase 2 trial demonstrated continued safety, including no antidrug antibodies and efficacy of Mim8 in participants with hemophilia A with or without FVIII inhibitors [58]. An extension study is underway.

TFPI is a key regulator of the extrinsic pathway of thrombin generation and acts to downregulate TF. Inhibition of TFPI serves to promote coagulation and enhance thrombin generation. Two anti-TFPI molecules are in clinical development, concizumab (approved in Canada) and marstacimab (approved in the USA), while the development of a third, befovacimab, has been terminated due to thromboses found in early clinical trials. These molecules are effective in people with hemophilia A and B with and without inhibitors and have the advantage of subcutaneous bioavailability. A thrombotic risk was identified in early-stage clinical trials of concizumab. A risk mitigation strategy appears to have resolved this issue. Antidrug antibodies were observed in 23 of 112 recipients of marstacimab. These antidrug antibodies were not inhibitory. Fitusiran is an antithrombin antisense oligonucleotide that knocks down antithrombin expression [59]. Safety concerns with this molecule include the risk of venous thromboembolism, elevation of transaminase enzymes, and unexplained biliary events, which appear to be related to antisense oligonucleotide technology rather than antithrombin knockdown [60]. Two other molecules in early clinical development are Serpin [61,62] and SR604 [63,64].

Eptacog beta is a novel rFVIIa molecule expressed in the mammary glands of transgenic rabbits. It demonstrates improved pharmacokinetics compared with eptacog alpha and, similar to eptacog alpha, catalyzes the conversion of prothrombin to thrombin by a TF-dependent mechanism. Unlike eptacog alpha, it also has novel mechanisms of action that serve to potentiate its prothrombotic effects. This molecule demonstrates a 40% greater affinity for the platelet membrane, thereby enhancing the amplification phase of thrombin generation. It competes with protein C for binding to the endothelial cell protein C receptor (EPCR), preventing FVa inactivation by activated protein C. In addition, eptacog beta binds to endothelial cells independent of EPCR to induce EPCR- and protease-activated receptor 1-dependent release of extracellular vesicles from endothelial cells, further enhancing thrombin generation. A robust clinical development program has been completed and was recently reviewed [65]. Program for the Evaluation of Recombinant Factor Seven Efficacy by Prospective Clinical Trial (PERSEPT) 1 demonstrated the safety and efficacy of eptacog beta in people with hemophilia A or B and inhibitors who were 12 years of age and older. PERSEPT 2 demonstrated the safety and efficacy of eptacog beta in people with hemophilia A or B with inhibitors younger than 12 years of age. PERSEPT 3 demonstrated the safety and efficacy of eptacog beta in people with hemophilia A or B and inhibitors of any age undergoing major or minor procedures. A postmarketing surveillance study is ongoing [66].

Since the genes encoding FVIII [67] and FIX [68] were cloned over 4 decades ago, the goal has been to develop a safe and effective gene therapy that is predictable and durable to treat people with hemophilia A or B [69]. Hemophilia was identified as an ideal test case for gene therapy in that it is a monogenic disease with a clearly defined phenotype and a wide therapeutic window [70]. Furthermore, small and large animal models exist in which prospective gene therapies can be tested and refined [71]. In 2022, the European Medicines Agency (EMA) granted conditional marketing authorization to valoctocogene roxaparvovec (AAV5-hFVIII-SQ) (BioMarin Pharmaceutical Inc), an adeno-associated virus 5 gene therapy to treat hemophilia A [72], and the FDA granted approval in 2023 to etranacogene dezaparvovec-drlb (AAV5-FIX Padua) (CSL Behring) to treat hemophilia B [73], followed by conditional marketing authorization by the EMA in early 2023. A second gene therapy for people with hemophilia B, fidanacogene elaparvovec-dkzt (Pfizer Inc), received marketing authorization in Europe in December 2023 and the USA in April 2024 [74]. Approval of these gene therapies was based on efficacy data that demonstrated reductions in the rates of bleeding compared with prior therapy with factor replacement products, factor concentration usage, and acceptable safety profiles [[74], [75], [76], [77]]. The year-over-year FVIII expression levels following valoctocogene roxaparvovec administration demonstrate declining expression with the most recent data presented at the 2024 European Association for Haemophilia and Allied Disorders (EAHAD) Congress [78]. The outlook for people with hemophilia B who have received etranacogene dezaparvovec-drlb is considerably more favorable with durable FIX activity at therapeutic levels 3 years after receiving the product [76]. This difference between FVIII and FIX expression may be related to the complexity of the FVIII protein compared with FIX and/or the natural cell of synthesis of the 2 proteins (FVIII is naturally made in sinusoidal endothelial cells while FIX is synthesized in hepatocytes, the cell target for both transgenes).

The ultimate goal of people with hemophilia is to be cured of the disease. However, people living with hemophilia and medical experts differ on the definition of a cure, and no universally accepted definition exists. Amelioration of symptoms and freedom from constant thoughts about the disease [79] is frequently noted as a definition of a cure; however, it should be noted that none of the currently approved products nor any in clinical development will prevent the genetic transmission of the trait to offspring.

## 4 Comprehensive Interdisciplinary Care

A clinic for people with inherited bleeding disorders was established at the University of Sydney Medical School and Royal Prince Alfred Hospital in 1957 by Professor Blackburn and provided comprehensive care for about 300 patients, about half of whom were affected by hemophilia [80]. In the United Kingdom [81] and Canada [82], comprehensive care was also delivered through specialized centers in addition to specialized boarding schools in France [83] and the United Kingdom [84]. The Hemophilia Act of 1973 in the USA allowed the establishment of comprehensive care centers and authorized federal funding to support them. Amidst the growing complexity of the various treatment options, hemophilia treatment centers (HTCs) are vital in personalizing care through an interdisciplinary approach. By relying on the expertise of specialists such as hematologists, nurses, social workers, physical therapists, and other healthcare professionals, HTC providers create individualized treatment plans that address each patient’s unique needs. This integration of expertise tackles the medical, psychological, and social aspects of hemophilia and other bleeding disorders, ultimately enhancing patient outcomes and HRQoL [3].

The introduction of gene therapy into the treatment choices for people with hemophilia has been long awaited [85]. Discussing gene therapy as part of the integrated, comprehensive care model is considered best practice [86], including the use of standardized language to facilitate effective communication between people with hemophilia and their healthcare providers [87]. As part of their accreditation process, the EAHAD and European Haemophilia Consortium have developed updated guidelines [88], including requirements related to the introduction of the hub-and-spoke model for the delivery of gene therapy [89]. In addition, EAHAD has developed a framework to collect standardized information following the administration of gene therapy [90] consistent with the World Gene Therapy Registry of the WFH [91,92].

Irrespective of the treatment options available to people with hemophilia in a particular country, they should be engaged in the choice of their therapy whenever feasible [93]. Including people with hemophilia as lived experience experts (LEEs) in treatment choices by means of shared decision-making (SDM) stands endorsed by experts and must be central to care provided at HTCs. This collaborative approach involves people with hemophilia and their families in the decision-making process, aligning treatment options and regimens with their preferences, values, and lifestyles. No matter where they live in the world, engaging people with hemophilia in their care empowers them to make informed treatment decisions, fosters trust, and increases adherence to treatment plans, which also contributes to improved health outcomes and HRQoL [94,95].

Education focusing on different therapeutic options, prevention, and safe and effective treatment of bleeding events and joint health, among other things, is another critical component of HTC care. The early recognition and prompt treatment of bleeding through self-infusion is perhaps more important than ever before. The “wait and see” approach supported by a shift in treatment paradigms associated with the emergence of novel therapeutics may prove to be detrimental in the future [96]. Even with the best treatments available, recurrent hemarthrosis, which can lead to chronic pain, reduced mobility, and hemarthropathy, is still possible [39].

## 5 Remaining Gaps in Care for People with Hemophilia A or B

Many open questions exist for people with hemophilia, their clinicians, and governments in the countries where they live (Table 2). Increasing the identification and diagnosis of people with hemophilia in all countries, but in particular in L/MIC, will require ongoing education of the population as to what bleeding disorders are and how they manifest, as well as education of healthcare professionals to increase their ability to recognize and then diagnose and refer someone suspected to have hemophilia or another bleeding disorder for appropriate care. Srivastava [97] has suggested that a new paradigm be adopted in which validated bleeding assessment tools be used in primary care settings or even self-administered to identify people at risk of having a bleeding disorder, and then next-generation sequencing be employed rather than cumbersome tests currently used in clinical coagulation laboratories around the world. This approach would require an investment to educate and train people on the use of bleeding assessment tools, but it could be cost-saving to the government. Other novel technologies, such as luminescent caged carbohydrate substrate-based microassays [98] or dielectric spectroscopy microsensors [99], are in development and might be available at low cost, benefiting L/MIC. Standardization of the thrombin generation assay would be beneficial if it were allowed to be used in clinical laboratories [100].

Gaps in care and treatment also exist as the quest for equitable outcomes for all people with hemophilia remains a goal not only in L/MIC but all countries. For example, in the USA, people with hemophilia living in rural communities experience delays in diagnosis and decreased access to care [101]. Once diagnosed, there are numerous options currently available to treat people with hemophilia A and B, which are summarized in Table 1. Not all products may be available in all countries, and in fact, some L/MIC rely on humanitarian aid from the WFH to sustain prophylaxis and allow surgery to proceed in a small number of people with hemophilia [102]. Opportunities exist to optimize the use of available molecules, including the EHL FVIII and FIX products and eptacog beta. Recently, there has been considerable interest in personalizing therapy with emicizumab using lower doses of the drug [[103], [104], [105], [106], [107]], which may provide more equitable access to prophylaxis in L/MIC [108]. The regulatory authorization of efanesoctocog alfa [33], a highly modified rFVIII with a clinically significantly prolonged half-life, and the first drug, concizumab [109], targeting TFPI in 2023, was followed by a second, marstacimab in 2024 [110], each added to the options available for people with hemophilia, but only in countries where these innovations are affordable [111]. Most new treatments, including many in development, target increased thrombin generation rather than replacing the missing FVIII or FIX activity [6,112]. Unfortunately, thrombin generation assays lack standardization and are not widely available except in some research laboratories [100]. A more comprehensive understanding of the importance of preanalytical conditions and the reliability and consistency of the testing procedures precludes implementation in clinical settings, which would be useful to personalize treatments for specific conditions faced by people with hemophilia [113]. Research to understand how to standardize and deploy the thrombin generation assay in the clinic is an area of unmet need (Table 2).

Understanding the educational gaps between people with hemophilia and their caregivers is an area of interest. The use of simulation is commonplace in medical education but has not been used widely in patient education. How might simulation be used to support patient education? Closing these and other educational gaps and gaining a better understanding of the biology of bleeding disorders, especially in women, remain high-priority areas.

In 2021, the NBDF, previously the National Hemophilia Foundation, conducted the Research State of the Science with extensive input from community members, including LEEs—those living with or caring for people with hemophilia and other inherited bleeding disorders [114]. Individual consultations, focus groups, and community surveys informed the recommendations of several working groups charged with identifying the issues most important to LEEs, as well as to clinicians, researchers, government and industry partners, and other community members. These expert multidisciplinary working groups, including one focused on gaps in care for hemophilia, were assembled and worked to understand the key areas of need, define and prioritize research questions based on feasibility, impact, and risk, and identify the resources and infrastructure needed to address them [115]. A subsequent initiative, the National Research Blueprint (NRB), carried out from 2022 to 2024, focused on refining the latter (https://www.bleeding.org/research/national-research-blueprint).

Specifically, the NRB was a community-wide effort to redefine research in bleeding disorders through a comprehensive, integrated, and collaborative national research strategy. It stipulates that the lived experience must drive and influence research while promoting equity, diversity, and inclusion. A draft of the initial NRB was presented at a summit in early 2024, during which community input was again sought. Manuscripts describing the process are forthcoming. The integration of LEEs was key to driving focus and maintaining objectivity [116].

Disparities prevalent in people with hemophilia include underrepresentation and underdiagnosis of women, girls, and people with the propensity to menstruate, racial and ethnic disparities, and socioeconomic barriers to care. NBDF’s NRB also seeks to address some of these questions (Baldwin et al.; [117]). The voice of the LEE is critical, as is the objective. Table 2 shows an overview of some of the issues identified by LEEs that might be addressed in the NRB, provided there is adequate funding to support the infrastructure and a well-trained and capable workforce to carry out the research and provide clinical care implementing the advances [118]. Although these recommendations focus on the USA, many are applicable to others around the world.

## 6 Conclusions

The past 7 decades have brought unparalleled innovations in treatment regimens and products for those impacted by hemophilia; however, achieving a hemophilia-free state [79] remains an aspiration for most of the world’s population living with hemophilia. This is particularly true for L/MIC, where large treatment and outcome disparities exist [119,120]. This hemophilia-free mindset has been espoused as a goal for treatment [121]; however, even with the currently available options to treat people with hemophilia, it may not be achievable. Bleeding may be ameliorated (“bleed-free”), but existing morbidities, including joint disease (arthropathy) and pain, will not be eliminated, and genetic transmission remains a possibility.

Despite the widespread implementation of prophylaxis, people with hemophilia continue to experience bleeding, including into their joints, with resulting arthropathy, disability, and decreased HRQoL [39,122]. Real-world data from observational, noninterventional, prospective, multicenter studies of FVIII prophylaxis in all severity forms of hemophilia A demonstrate that only 34% to 56% of people with hemophilia can achieve a state of no bleeding with FVIII prophylaxis [34]. For instance, across the emicizumab clinical development program (HAVEN 1-4), 46% of children, adolescents, and adults with hemophilia A, with or without inhibitors directed to FVIII, experienced no bleeding [42].

Therefore, research and future investigations must focus first on preventing all bleeding, especially joint bleeding, including subclinical joint bleeding [97]. New approaches to detect joint bleeding are needed. Deployment of and education on the use of point-of-care musculoskeletal ultrasound for people with hemophilia will be critical to ensure early detection of bleeding [123,124], as will the development of biochemical markers of joint bleeding capable of detecting trace amounts of blood not perceived by people with hemophilia [125]. The use of new products must be accessible, optimized, and personalized to achieve the best possible outcomes for everyone with hemophilia, including women and those with nonsevere disease. The goal is health equity, inclusion, and access to care for all. As treatment continues to evolve, comprehensive care addressing psychosocial aspects, including mental health, will be key to ensuring optimal outcomes for people with hemophilia.

The expanding landscape of treatment options for people with hemophilia and the resulting paradigm shifts emphasize the indisputable increased need for education, not just for patients but also for clinicians. To ensure optimal SDM, education initiatives should focus on understanding options and unique characteristics of current and investigational treatment modalities, identifying and aggressively treating breakthrough bleeding, recognizing when to change regimens if the current one is not meeting expectations, and, importantly, self-infusion instructions, even for people with hemophilia on subcutaneous therapy and postgene therapy, as needed.

By focusing on personalized treatment regimens using SDM and rigorous education of people with hemophilia and their healthcare providers, HTCs ensure holistic and patient-centered care for people with hemophilia and other bleeding disorders. This approach not only addresses immediate health needs but also promotes long-term well-being and improved HRQoL, which must be afforded to all people with hemophilia, irrespective of where they live in the world.
